# Erythroderma: A clinical study of 97 cases

**DOI:** 10.1186/1471-5945-5-5

**Published:** 2005-05-09

**Authors:** Maryam Akhyani, Zahra S Ghodsi, Siavash Toosi, Hossein Dabbaghian

**Affiliations:** 1 Department of Dermatology, Tehran University of Medical Sciences, Razi Hospital, Vahdate-Eslami Sq. 11966, Tehran, Iran

## Abstract

**Background:**

Erythroderma is a rare skin disorder that may be caused by a variety of underlying dermatoses, infections, systemic diseases and drugs.

**Methods:**

We reviewed the clinical, laboratory and biopsy material of 97 patients diagnosed with erythroderma who were treated in our department over a 6-year period (1996 through 2002).

**Results:**

The male-female ratio was 1.85:1. The mean age at diagnosis was 46.2 years. The most common causative factors were dermatoses (59.7%), followed by drug reactions (21.6%), malignancies (11.3%) and idiopathic causes (7.2%). Carbamazepine was the most common drug (57.1%). The best clinicopathologic correlation was found in cutaneous T-cell lymphoma and pityriasis rubra pilaris related erythroderma. Apart from scaling and erythema that were present in all patients, pruritus was the most common finding (97.5%), followed by fever (33.6%), lymphadenopathy (21.3%), edema (14.4%) and hyperkeratosis (7.2%).

**Conclusion:**

This study outlines that underlying etiologic factors of erythroderma may show geographic variations. Our series had a high percentage of erythroderma secondary to preexisting dermatoses and a low percentage of idiopathic cases. There was no HIV-infected patient among our series based on multiple serum antibody tests. The clinical features of erythroderma were identical, irrespective of the etiology. The onset of the disease was usually insidious except in drug-induced erythroderma, where it was acute. The group associated with the best prognosis was that related to drugs.

## Background

Erythroderma or exfoliative dermatitis is a rare skin disorder that may be the result of many different causes. It represents an extreme state of skin irritation involving the whole or most of the skin surface. Because most patients are elderly and skin involvement is widespread, the disease implies an important risk to the life of the patient [1]. Hasan and Jansen estimated the annual incidence of erythroderma to be 1 to 2 per 100,000 patients [2].Sehgal and Strivasta recorded the incidence of erythroderma in a large prospective study from the Indian subcontinent as 35 per 100,000 dermatologic outpatients [3]. The causative factors can be grouped as previous dermatoses, drug reactions, malignancies systemic diseases, infections and idiopathic disorders. The four more common causes of idiopathic protracted erythroderma are probably atopic dermatitis of the elderly, intake of drugs overlooked by the patient, pre-lymphomatous eruptions and occult malignancies [1,4]. Histopathology can help identify the cause of erythroderma in up to 50% of cases, particularly by multiple skin biopsies [5]. Many chronic dermatoses may be histologically indistinguishable in erythrodermic patients [6].

In Pakistan, Pal and Haroon have studied the features of erythroderma in 90 patients and etiologically found preexisting dermatoses as the most frequent cause of erythroderma [7]. To date there are no published studies on the frequency of underlying causes of erythroderma from Middle East. In order to delineate the salient features of erythroderma in our region, we have reviewed the cases of erythroderma examined and treated in our institution between 1996 and 2002 (six years). Finally our data are discussed and compared with other earlier series that are taken from Western and European populations as well as from other Asian countries.

## Methods

We defined erythroderma as generalized erythema of the skin (more than 90% of the body surface area) accompanied by a variable degree of scaling [4].

The population covered by our institute is difficult to establish, because it is a tertiary reference hospital that receives patients from distant areas. A coverage of 5,000,000 is an approximate figure. Due to the risk that erythroderma implies for the patient's life and to study the cause in each patient, we always treat them as inpatients. The records of patients who were discharged with a diagnosis of erythroderma in the period from 1996 to 2002 (six years) were carefully reviewed and the following data recorded for all the patients: personal data, history of skin diseases, past medical history, drug history, previous episodes of erythroderma, onset of erythroderma (acute or insidious), clinical data during the episode (scaling, pruritus, lymphadenopathy, visceral enlargement, hyperkeratosis, mucosal involvement and edema). Laboratory investigations including complete hematological parameters, erythrocyte sedimentation rate, serum protein levels, liver and kidney function tests, serum electrolytes, urine microscopy, stool exam for occult blood, serum markers for viral hepatitis B and C and HIV antibody testing, microscopy for scabies mite and fungus, electrocardiography, and chest radiography were performed for all the patients as a routine in the dermatology ward of our hospital. Disease-specific investigations such as skin biopsy, lymph node biopsy, immunophenotyping, flow-cytometry, patch test and work-up for occult malignancy were performed in special cases as indicated.

## Results

In the six year study period, erythroderma was found in 97 patients. Sixty three (64.9%) patients were male and thirty four (35.1%) were female, that means men outnumbered women in a proportion of 1.85:1. The mean age of the onset of erythroderma was 46.2 ± 20.03 (SD) years (range, 8 through 90 years). There was no significant difference in the age of onset between male and female groups using independent T-test. All the patients in this series were Caucasian.

All the patients had some degree of scaling. Ninety-four (97.5%) patients had pruritis. Thirty two (33.6 %) patients had a fever (temperature, 38°c or higher) during the episode and lymphadenopathy was found in 20 (21.3%) patients. Visceral enlargement was found in 4 (4%) patients. Edema, hyperkeratosis and mucosal involvement were found in 14 (14.4%), 7 (7.2%) and 1 (1%) patients respectively.

Histopathologic examination was performed in 81 (83.5%) of the 97 patients. Skin biopsy was performed in all the cases classified as malignancy and idiopathic. A skin biopsy was not performed in some of the cases because the cause of erythroderma was clear from the start (previous known dermatoses or treatment with some drug in the days before the appearance of erythroderma).

Final diagnosis was the result of evaluation of the clinical, biochemical and histological findings and of the evolution of erythroderma in each individual patient. The patients were divided into four etiologic groups: (1) Previos dermatoses (58 patients, 59.8%): psoriasis, 27 (27.8%); atopic dermatitis, 13 (13.4%); pityriasis rubra pilaris, 8 (8.2%); seborrheic dermatitis, 2 (2.1%); contact dermatitis, 3 (3.1%); actinic reticulosis, 1 (1%); scabies, 1(1%); bullous ichthyosiformis, 1 (1%); pemphigus foliaceus, 1 (1%) and senile xerosis, 1 (1%), (2) Malignancies (11 patients, 11.3%): Sezary syndrome, 2 (2.1%); mycosis fungoides, 8 (8.2%); lung cancer, 1 (1%), (3) Drug reactions (21 patients, 21.6%): carbamazepine, 12 (57.1%); phenytoin, 3 (14.3%); phenobarbital, 2 (9.5%); lithium, 1 (4.8%); penicillin,1 (4.8%); vancomycin, 1 (4.8%) and cotrimoxazole, 1 (4.8%) and (4) Idiopathic or undetermined: seven patients (7.2%). Relationship between a drug and erythroderma was established from the antecedent of intake of the incriminating drug in the days preceding the onset of erythroderma and clearing of the manifestations following withdrawal of the drug. Mild anemia, increased erythrocyte sedimentation rate, leukocytosis and hypoalbuminemia were common findings but nothing remarkable was found in the laboratory tests. None of the patients were HIV-positive based on the multiple serum antibody tests. All the patients in the idiopathic group as well as those with seborrheic dermatitis, actinic reticuloid and Sezary syndrome were males. All the three patients with contact dermatitis as a cause of their erythroderma were female. There was no statisticaly significant difference in the frequency of causes of erythroderma among men and women.

The onset of erythroderma was generalized in 23 (23.7%) patients. In others the initial site of involvement of erythroderma was head and neck in 20 (20.6%), upper or lower limbs in 18 (18.5%), trunk in 11 (11.3%), genitalia in one (1%), flexural regions in five (5.2%) and extensor areas in 19 (19.6%) patients. On follow-up, only one patient with lung cancer died due to erythroderma or its underlying causes. None of the patients with drug reactions and pityriasis rubra pilaris relapsed. With therapy, erythroderma improved in all the patients with mycosis fungoides and Sezary syndrome but relapse occured in all of them at least once again. All the patients with psoriasis was hospitalized for the second time during the study period.

## Discussion

The approach to patients with erythroderma depends on their previous dermatologic history. Patients with dermatologic disorders recalcitrant to therapy may develop erythroderma during a flare up. In such cases, the etiologic diagnosis is easy to establish, otherwise, erythroderma remains a diagnostic challenge. The clinical features of erythroderma are non-specific and certain clues such as scaling or pruritis could not be related to any specific cause. Erythroderma of long duration may cause hair loss or nail dystrophy regardless of its origin, so these changes are also non-specific. In erythrodermic patients clinicopathologic correlation is usually poor, because the specific cutaneous changes of dermatoses or drug reacions are obscured by the non-specific changes induced by the inflammatory process of erythroderma [1]. In a patient without history of dermatologic diseases and who denies having recently taken any medication, the diagnosis is more difficult and it is of great importance to perform skin biopsies in such cases although the histologic picture shows either a subacute or chronic dermatitis and psoriasiform reaction. Thus each case of undetermined origin requires thorough histologic examination through multiple skin biopsies and a lymph node biopsy to rule out lymphoma. A drawback in our study was that it was a retrospective survey and, therefore, we were not able to verify the diagnosis ourselves. In spite of these drawbacks we believe that our data on causes of erythroderma would add to the already existing literature on erythroderma.

In our series the mean age of onset was in fifth decade and men outnumbered wemon. Such findings are in accordance with many other studies [1,7-10]. The best clinicopathologic correlation was found in mycosis fungoides and pityriasis rubra pilaris. Previously, Botella-Estrada et al have also reported similar findings [1].

Like many other series [2,3,9], diffuse scaling and pruritus was found in almost all of our patients. Although we examined each patient in several occasions, we found a lower percentage of lymphadenopathy, visceral enlargement, edema and mucosal involvement in comparison with Pal and Haroon's series [7]. Many drugs can cause erythroderma. Among the more commonly implicated are pyrazalone derivatives, carbamazepine, hydantoin derivatives, cimetidine, lithium salts and gold salts [9,11]. According to our findings, the agents of greatest erythroderma-inducing potential are carbamazepine, phenytoin and phenobarbital. The drugs responsible for erythroderma in our series have been previously incriminated as a cause of this disorder in literature [9]. Carbamazepine was the drug most frequently quoted in our cases, with 12 of the 21 cases (57.1%) related to this medication. This drug has been mentioned as a less frequent cause of erythroderma in other series [1,2,12,13]. Thus carbamazepine as a frequent cause of erythroderma in the present population warrants particular attention and may be due to a genetic sensitivity to this drug or a high rate of its prescription.

Surprisingly, in spite of the fact that, allupurinol is prescribed frequently in this country we observed no erythroderma related to this drug. Allopurinol has been mentioned as one of the most common causes of drugs inducing erythroderma in some other recent series [1,2,12,13].

Comparison of the etiologic groups among the recent previous series and our own is given in table 1. Our series has a high percentage of erythroderma secondary to preexisting dermatoses that are mentioned as the most common cause of adult erythroderma in the majority of studies [1-4,9,13,14]. Drug reactions were the most common cause of erythroderma in the HIV-positive patietns in one report [8]. The percentage of cases in which no underlying disease is demonstrable diminishes with the thoroughness of investigation and the duration of observation, but in any series of cases it is rarely below 10% [1,2,4,12-15]. In our cases we found a particularly low percentage of idiopathic cases.

**Table 1:** classification of patients with erythroderma by cause in some previous publications and in the present series.

	Relative incidence,(%)							
Causes	Sigurdsson *et al*^14^, 1996	Pal and Haroon^7^,1998	Nicolis and Helwig^15^, 1973	Hasan and jansan^2^, 1983	Botellaestrada *et al*^1^, 1994	Sehgal and strivastava^12^,1986	King *et al*^13^, 1986	The present series 2004

Preexisting dermatoses	53	74.4	25	42	62.5	52.5	30	57.9
Drug reacions	5	5.5	42	22	16	24.7	34	21.6
malignancies	13	5.5	21	4	12.5	0	20	11.3
Idiopathic	26	14.6	12	32	9	22.5	16	7.2

Eight (8.2%) of our patients were diagnosed as pityriasis rubra pilaris which is uncommonly reported as a cause of erythroderma in other studies [1,2,12,13]. Pal and Haroon reported pityriasis rubra pilaris as a causative factor of erythroderma in 2.2% of their patients [7]. As a result, we concluded that there might be a higher frequency of pityriasis rubra pilaris or a greater tendency to its generalization in our environment. Psoriasis was the most common underlying cause, which is in accordance with Pakistanian and Indian series [7,3].

The onset of erythroderma was usually insidious except in drug-induced cases, where it was abrupt and florid. This is in accordance with three other studies [1-3], which reported gradual onset in most cases. In Pal and Haroon's series, erythroderma started with an acute onset in more than two-thirds of the patients which is assumed to be related to the injudicious use of medication [7]. In our patients, the group associated with the best prognosis was that related to drugs such findings have been observed in literature [9].

Because erythroderma is occasionally associated with internal malignancies, even patients with previous history of known dermatoses whose clinicopathologic features are inconclusive, should be investigated carefully to rule out malignant neoplastic causes.

We observed no remarkable laboratory results in our patients. this finding was similarly reported by Haroon and Pal [7]. There was no HIV-infected patient in the present series. Erythroerma is reported frequently due to different dermatoses or drug reactions in HIV-positive patients. In one series reported by Morar et al, a large proportion of erythrodermic patients were HIV-positive but they did not have a significant increase in he number of episodes of erythroderma. It is concluded that in the young black patients erythroderma may be a marker for HIV infection [8]. In harmony with its low frequency, HIV seems to be a lesser threat in our community.

In initial documented studies, the recorded death rate due to erythroderma varied from 18 to 64% [9]. On follow-up, we found only one death related to erythroderma or its underlying causes, which was the patient with lung cancer. Our findings support Hassan and Jansen's view [2] that erythroderma does not pose a significant risk to the patient's life.

This study outlines that some important features of erythroderma may show geographic variations.

## Conclusion

Our series had a high percentage of erythroderma secondary to preexisting dermatoses and a low percentage of idiopathic cases. The clinical features of erythroderma were identical, irrespective of the etiology. The onset of the disease was usually insidious except in drug-induced erythroderma, where it was acute. This study outlines that underlying etiologic factors of erythroderma may show geographic variations.

## Abbreviations

HIV: Human Immunodeficiency virus

## Competing interests

The author(s) declare that they have no competing interests.

## Authors' contributions

All authors contributed equally in the study design, literature search, data analysis and manuscript preparation. All authors read and approved the final manuscript.

## Pre-publication history

The pre-publication history for this paper can be accessed here:


